# Targeting ADAR1 suppresses progression and peritoneal metastasis of gastric cancer through Wnt / β-catenin pathway

**DOI:** 10.7150/jca.61031

**Published:** 2021-10-28

**Authors:** Zhiyong Li, Yunning Huang, Yuanyi Xu, Xiaofei Wang, Honghong Wang, Shuai Zhao, Han Liu, Guangfu Yu, Xiangming Che

**Affiliations:** 1 General Surgery Department, The First Affiliated Hospital of Xi'an Jiaotong University, Xi'an 710061, Shaanxi, China.; 2 Department of Gastrointestinal Surgery, People's Hospital of Ningxia Hui Autonomous Region, Yinchuan 750002, Ningxia, China.; 3 Department of Pathology, School of Basic Medicine, Ningxia Medical University, Yinchuan 750004, Ningxia, China.; 4 Department of Pathology, North China University of Science and Technology Affiliated Hospital, Tangshan 063000, Hebei, China.; 5 Department of Pathology, People's Hospital of Ningxia Hui Autonomous Region, Yinchuan 750002, Ningxia, China.; 6 Digestive Endoscopy Center, People's Hospital of Ningxia Hui Autonomous Region, Yinchuan 750002, Ningxia, China.; 7 Department of Surgery, School of Clinical Medicine, Ningxia Medical University, Yinchuan 750004, Ningxia, China.

**Keywords:** ADAR1, gastric cancer, peritoneal metastasis, Wnt / β-catenin pathway, CALR

## Abstract

**Objective:** Peritoneal metastasis frequently occurs in advanced gastric cancer, which is typically not eligible for radical surgery. Here, this study observed the function and regulatory mechanism of ADAR1 in peritoneal metastasis of gastric cancer.

**Methods:** ADAR1, CALR and β-catenin proteins were detected in normal mucosa, primary gastric cancer, metastatic lymph node and metastatic omentum tissues by immunohistochemistry, western blot, and immunofluorescence. After silencing ADAR1 by siADAR1, the effect and mechanism of ADAR1 on gastric cancer metastasis were observed in nude mouse models of gastric cancer with peritoneal metastasis as well as HGC-27 and AGS gastric cancer cells.

**Result:** Our results showed that ADAR1 was significantly up-regulated in gastric cancer, metastatic lymph node and metastatic omentum tissues. Its up-regulation was significantly correlated to lymph node metastasis and peritoneal metastasis. Silencing ADAR1 significantly reduced the volume of peritoneal metastatic tumors and weakened oncogene CALR expression, Wnt / β-catenin pathway and epithelial-mesenchymal transition (EMT) process *in vivo*. Furthermore, ADAR1 knockdown distinctly suppressed cell viability, colony formation and migration of HGC-27 and AGS cells and ameliorated the effects of Wnt pathway activator on tumor progression. The similar findings were investigated when treated with ADAR1 inhibitor 8-Azaadenosine.

**Conclusion:** Collectively, this study identified a novel oncogenic function of ADAR1 in peritoneal metastasis of gastric cancer via Wnt / β-catenin pathway. Hence, ADAR1 could be a novel marker and therapeutic target against gastric cancer metastasis.

## Introduction

Gastric cancer ranks fifth in incidence and third in cancer-related mortality globally among all cancers 1. Approximately 90% of gastric cancer is adenocarcinomas 2. Surgical resection remains the only treatment strategy to cure this malignancy 3. However, even for patients at the early stage, distant metastasis rate within five years stays at a high-level following surgery. The peritoneum is the most common metastatic site following surgery 4. About 66% of advanced patients experience peritoneal metastasis 5. Approximately, one third of patients are firstly diagnosed at the late stage. Once peritoneal metastasis occurs, the median survival time of patients is no more than 4 months 6. This malignancy is highly heterogeneous at molecular and phenotypical levels. Epithelial-mesenchymal transition (EMT) is involved in gastric cancer metastasis 7. This process may be mediated by Wnt / β-catenin pathway 8. Wnt / β-catenin pathway is widely involved in proliferation, migration as well as metastasis of malignant tumors 9. Furthermore, this pathway could activate EMT process of gastric cancer 10. When the Wnt/β-catenin signaling pathway is activated, β-catenin accumulates in large quantities and is transported to the nucleus, thereby regulating the transcription of EMT-related genes 11. Hence, it is urgent to explore novel therapeutic targets against gastric cancer with peritoneal metastasis.

Adenosine deaminase 1 (ADAR1) that has 2 monomer forms ADAR1p110 and ADAR1p150 exerts a role on catalyzing the deamination of adenosine as well as converting adenosine (A) to inosine (I) 12. The oncogenic function of ADAR1 in gastric cancer has been reported in previous research. For example, Ma et al. reported that ADAR1 expression was elevated in gastric cancer than normal mucosa 13. Patients with high ADAR1 expression indicated dismal survival outcomes 14. ADAR1 up-regulation promoted gastric cancer progression through activation of mTOR/p70S6K axis 15. One of the roles of ADAR1 is to inhibit type I interferon (IFN) response. Jiang et al. found that ADAR1 suppressed IFN through miR-302a-mediated IRF9/STAT1 in gastric cancer 16. Here, this study found that ADAR1 was significantly correlated to gastric cancer metastasis. Targeting ADAR1 distinctly restrained peritoneal metastasis of gastric cancer, which was related to suppression of Wnt / β-catenin pathway. Our data were indicative of the potential of ADAR1 as a therapeutic target against gastric cancer metastasis.

## Materials and methods

### Clinical specimens

A total of 95 paraffin-embedded gastric cancer tissue sections were harvested from People's Hospital of Ningxia Hui Autonomous Region. All patients did not receive chemotherapy or radiotherapy before surgery. Gender, age, depth of invasion, lymph metastasis, TNM stage and peritoneal metastasis of each subject was gathered in** Table 1**. The research has been carried out in accordance with the World Medical Association Declaration of Helsinki. The research was approved by the Ethics Committee of People's Hospital of Ningxia Hui Autonomous Region (2019029).

### Immunohistochemistry

Paraffin sections were incubated with primary antibodies against ADAR1(1:200, sc-73408, SANTA CRUZ BIOTECHNOLOGY, USA), CALR (1:100; 27298-1-AP; Proteintech, China), β-catenin (1:200; 51067-2-AP; Proteintech, China), E-cadherin (1:150; ab231303; Abcam, USA), MMP2 (1:150; ab92536; Abcam, USA), MMP9(1:100; 10375-2-AP; Proteintech, China), TGFβ (1:100; 21898-1-AP; Proteintech, China) and Vimentin (1:100; 10366-1-AP; Proteintech, China) at 4 ℃ overnight. Then, the sections were incubated with secondary antibodies conjugated with HRP (#SPN-9100; ZSGB-BIO, Beijing, China) at room temperature for 1 h. The sections were stained through a DAB kit (#ZLI-9018; ZSGB-BIO, Beijing, China). The immunohistochemical scores were achieved by two experienced pathologists in a double-blinded manner, as previously described 17.

### Western blot

Protein samples were separated on SDS-PAGE. Following transference, the PDVF membrane was blocked by 0.5% skimmed milk at room temperature for 2 h and then incubated with primary antibodies against ADAR1 (1:400, sc-73408, SANTA CRUZ BIOTECHNOLOGY, USA ), CALR (1:1000; 27298-1-AP; Proteintech, China), β-catenin (1:1000; 51067-2-AP; Proteintech, China), E-cadherin (1:800; ab231303; Abcam, USA), MMP2 (1:1000; ab92536; Abcam, USA), MMP9 (1:500; 10375-2-AP; Proteintech, China) and Vimentin (1:1000; 10366-1-AP; Proteintech, China) and β-actin (1:3000; #ab8226; Abcam, USA) overnight at 4 °C. Afterwards, the sections were incubated with secondary antibodies (1:6000; ZB-2301 and ZB-2305; ZSGB-BIO, Beijing, China). The protein bands were visualized through ECL luminescence kit.

### Immunofluorescence

Tissue sections were incubated with primary antibodies labeled with fluorescent substance against ADAR1 (1:50, sc-73408, SANTA CRUZ BIOTECHNOLOGY, USA ), CALR (1:100; 27298-1-AP; Proteintech, China), β-catenin (1:100; 51067-2-AP; Proteintech, China), E-cadherin (1:150; ab231303; Abcam, USA) and Vimentin (1:1000; 10366-1-AP; Proteintech, China) overnight at 4 °C, followed by incubation with secondary antibodies Alexa Fluor® 488 Conjugate (1:100; #ZF-0512; ZSGB-BIO) as well as Alexa Fluor® 594 Conjugate (1:100; #ZF-0513; ZSGB-BIO) at room temperature lasting 2 h. The nuclear was counterstained by DAPI (ZLI-9557; ZSGB-BIO, China) for 5 min. The images were captured under a fluorescence microscope (BX61, Olympus, Japan).

### Xenograft nude mouse model

This animal experiment gained the approval of the Institutional Animal Care and Use Committee (IACUC) of People's Hospital of Ningxia Hui Autonomous Region (2019029). 4- or 5-week-old male BALB/c nude mice weighed 18-22 g were purchased from Beijing Vital River Laboratory Animal Technology Co., Ltd. (China; https://www.vitalriver.com/). 10 nude mice were randomly separated into control and siADAR1 groups. Following being anaesthetized with 0.5% pentobarbital sodium, mice were intraperitoneally administered by 5×10^6^ AGS cells that were resuspended with 300 μl serum-free medium. After two weeks, mice in siADAR1 group were treated with 25 mg / kg siADAR1 that was dissolved by corn oil through intraperitoneal injection twice one week. Meanwhile, control mice were treated with equal dose of corn oil. Following 4-week treatment, all mice were euthanized with excess pentobarbital sodium. Then, the tumor was removed from the abdominal cavity.

### Cell culture and transfection

Two human gastric cancer cell lines HGC-27 and AGS were purchased from Shanghai Zhongqiao Xinzhou Biotechnology Co., Ltd (http://www.zqxzbio.com/, Shanghai, China). These cells were maintained in RPMI-1640 medium plus 10% fetal bovine serum (11885084, Gibco, USA) in a humidified atmosphere with 5% CO_2_ at 37 °C. Three small interfering RNA (siRNA) oligonucleotides against ADAR1 (Sangon Biotech, Shanghai, China) were as follows: siADAR1#1: 5'-CAUCAAAUGCCUCAAAUAA-3' (sense), 5'-UAAAUGCUGUGCUAAUUGA-3' (antisense); siADAR1#2: 5'-GCCTCAAATAACATGGTAACC-3' (sense), 5'-CCATGAACCTCGATTTAAATT-3' (antisense); siADAR1#3: 5'-CCUUCUACAGUCAUGGCUUTT-3' (sense), 5'-AAGCCAUGACUGUAGAAGGTT-3' (antisense); siNC: 5'-UUCUCCGAACGUGUCACGUTT-3' (sense), antisense 5'-ACGUGACACGUUCGGAGAATT-3' (antisense). The oligonucleotides were transfected via Lipofectamine 2000 (#11668019, Invitrogen, USA) in line with manufacturer's instructions.

### Cell counting kit-8 (CCK-8) assay

Gastric cancer cells were treated with for 0, 5, 10, 20, 30, 40, 80 and 100 μM HLY78 (HY-122816, MedChemExpress, USA) for 48 h. The treated cells were inoculated onto 96-well plates for 24 h. Then, the cells were treated with 10 μl CCK-8 solution (Dojindo, Japan) for 2 h. The absorbance values were determined at 450 nm.

### Colony formation assay

Transfected cells were inoculated into 6-well plates (500 cells / well). They were cultured at 37 °C for 2 weeks. Afterwards, the colonies were fixed with 4% paraformaldehyde as well as stained with 0.5% crystal violet. Images were captured and cell colonies were counted.

### Transwell assay

Migration assay was carried out using 24-well transwell chambers (Millipore, Massachusetts, USA). 3 × 10^3^ transfected gastric cancer cells that were resuspended in serum-free RPMI-1640 medium were inoculated onto the upper chamber. Meanwhile, culture medium plus 10% FBS was added to the lower chamber. After incubation for 24 h, migrated cells were fixed with 4% paraformaldehyde and stained with 0.1% crystal violet. Under a microscopy (Olympus, Japan), the number of migrated cells in five fields / chamber was counted (200×).

### Wound healing assay

Transfected gastric cancer cells were seeded onto six-well plates. A 10-μL pipette tip was utilized for scratching wounds. At 0 and 48 h, images were obtained under a microscopy and wound distance was measured via ImageJ.

### Flow cytometry

Gastric cancer cells were treated with a series of concentrations of 8-Azaadenosine (0, 10, 20, 30 and 50 µM; HY-115686, MedChemExpress, USA) for 48 h. Apoptosis was detected via Annexin V-FITC/PI apoptosis detection kit following the manufacturer's instructions.

### Statistical analyses

Statistical analyses were presented using GraphPad Prism v8.0 (GraphPad, San Diego, CA) as well as SPSS v23.0 software (IBM SPSS, Armonk, NY, USA). Data were displayed as mean ± standard deviation. Relative ADAR1 expression was analyzed across gastric cancer and normal specimens from The Cancer Genome Atlas (TCGA) via the UALCAN web-portal (http://ualcan.path.uab.edu) 18. Furthermore, its expression was estimated in different subgroups on the basis of nodal metastasis status, tumor grade and individual cancer stage across gastric cancer samples. Comparisons between groups were analyzed by student's t test or one-way analysis of variance. Correlations between ADAR1 expression and clinicopathologic features were evaluated via Chi test. Spearson correlation between ADAR1 and CALR was assessed across gastric cancer samples using TCGA and GTEx data via the GEPIA web server (http://gepia2.cancer-pku.cn/) 19. P-value<0.05 was indicative of statistical significance.

## Results

### Up-regulation of ADAR1 is correlated with gastric cancer progression

**Figure 1:**
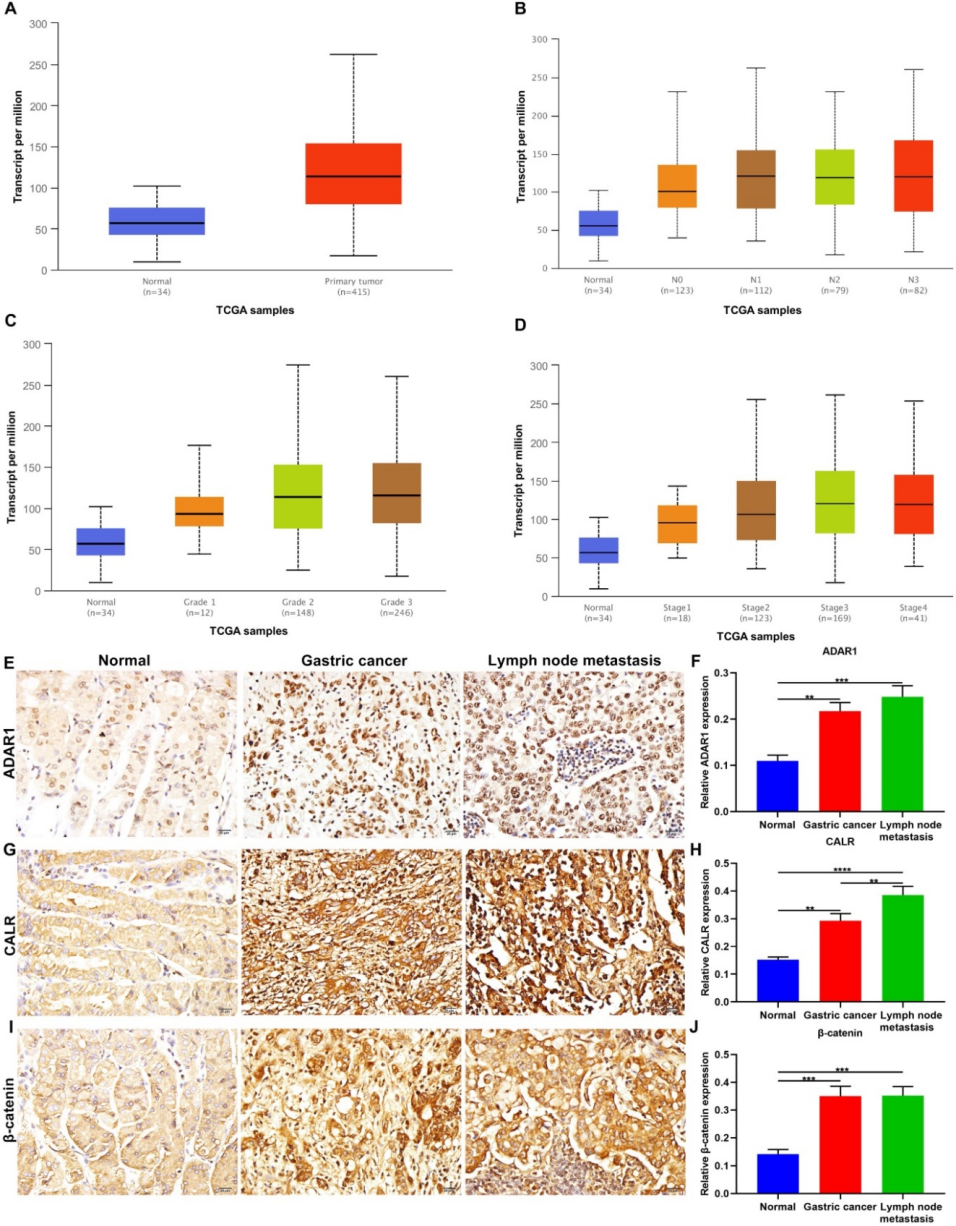
Up-regulation of ADAR1, CALR and β-catenin proteins in primary gastric cancer and metastatic lymph nodes. Assessment of relative ADAR1 expression in different subgroups according to (A) sample types, (B) nodal metastasis status, (C) tumor grade and (D) stage across gastric cancer samples from TCGA database. Immunohistochemistry for the expression of (E, F) ADAR1, (G, H) CALR and (I, J) β-catenin proteins in normal tissues, primary tumors, and metastatic lymph nodes. Bar = 20 μm. **p<0.01; ***p<0.001; ****p<0.0001.

ADAR1 expression was evaluated in gastric cancer and normal specimens from TCGA database. Our data showed the up-regulation of ADAR1 expression in tumor than normal tissues (p=1.62e-12; **Figure 1A**). Furthermore, we evaluated ADAR1 expression in different subgroups based on nodal metastasis status (**Figure 1B**), tumor grade (**Figure 1C**) and stage (**Figure 1D**) across gastric cancer samples. We found that ADAR1 expression exhibited positive correlations with the severity of gastric cancer. Here, we examined ADAR1 expression in 95 paraffin-embedded tissue sections. Compared to normal tissues, ADAR1 up-regulation was confirmed in primary tumors and metastatic lymph nodes (**Figure 1E, F**). This study evaluated the correlations between ADAR1 expression and clinicopathologic features across gastric cancer subjects. In **Table 1**, ADAR1 expression was significantly correlated to depth of invasion, TNM stage and peritoneal metastasis. CALR, an endoplasmic reticulum-resident protein, participates in various cellular processes 20. Here, higher CALR expression was found in primary tumors than normal tissues (**Figure 1G, H**). Also, its expression was distinctly higher in metastatic lymph nodes than primary tumors, indicating that CALR could be involved in malignant transformation of gastric cancer. Dysfunctional Wnt / β-catenin pathway is commonly investigated in gastric cancer. This study examined the expression of β-catenin, a key regulatory protein in this pathway. In **Figure 1I, J**, compared to normal tissues, β-catenin was significantly up-regulated in primary tumors as well as metastatic lymph nodes.

### Up-regulation of ADAR1 in gastric cancer peritoneal metastasis

**Figure 2:**
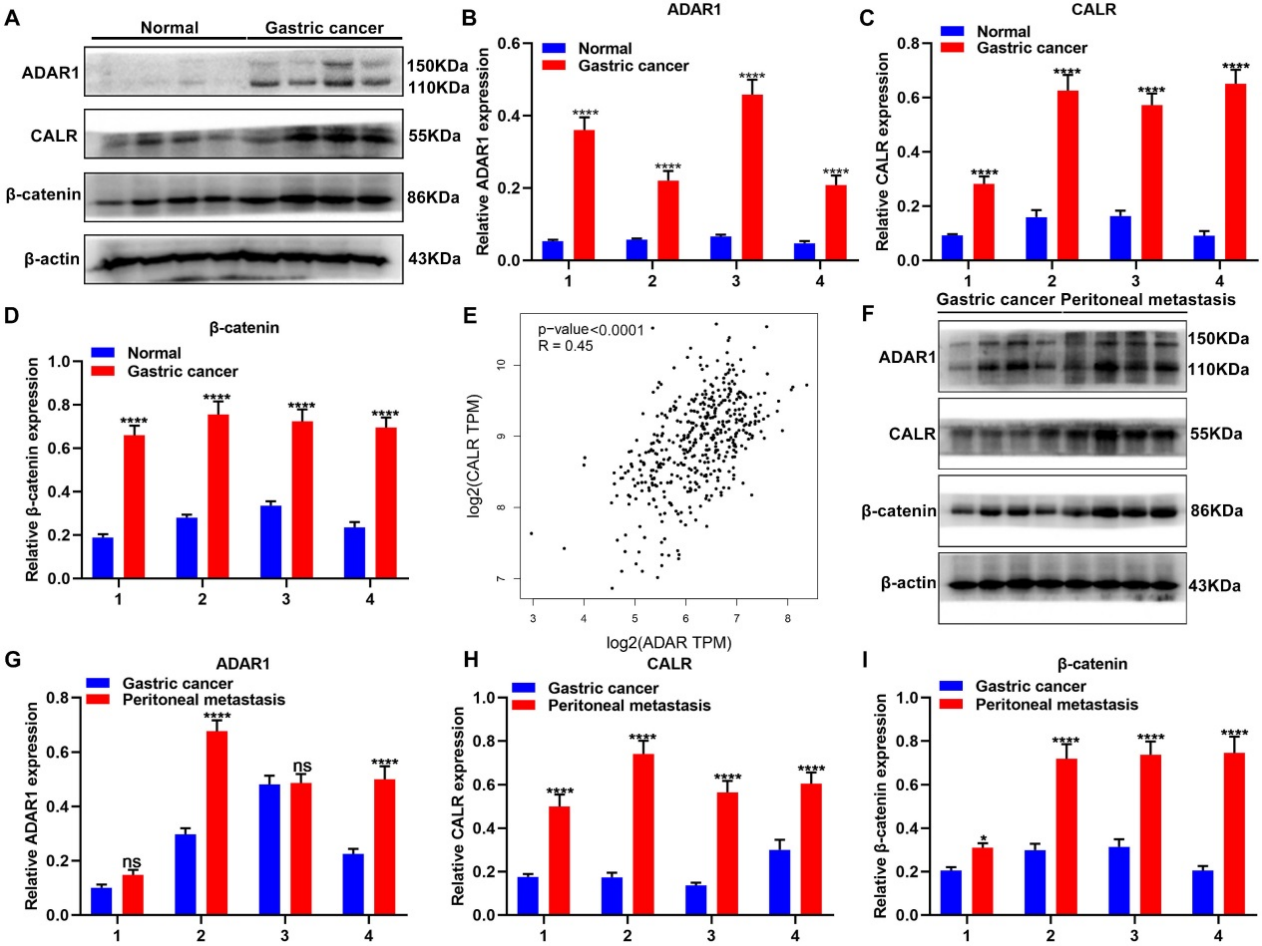
Overexpression of ADAR1, CALR and β-catenin proteins in primary gastric cancer and peritoneal metastasis tissues. (A) Western blot for expression of (B) ADAR1, (C) CALR and (D) β-catenin proteins in normal and gastric cancer tissues. (E) Correlation of ADAR1 with CALR across gastric cancer samples from TCGA database. (F) Western blot for expression of (G) ADAR1, (H) CALR and (I) β-catenin proteins in primary gastric cancer and peritoneal metastasis tissues. ****p<0.0001; ns: not significant.

Expression of ADAR1, CALR and β-catenin proteins was detected in 4 pairs of normal and gastric cancer tissues via western blot (**Figure 2A**). As a result, ADAR1, CALR and β-catenin were all significantly overexpressed in gastric cancer than normal tissues (**Figure 2B-D**). From TCGA database, CALR displayed a significantly positive correlation with ADAR1 across gastric cancer samples (**Figure 2E**). Peritoneal metastasis is commonly observed in advanced gastric cancer patients, which leads to undesirable survival outcomes 21. Here, this study detected ADAR1, CALR and β-catenin in primary gastric cancer and peritoneal metastasis tissues (**Figure 2F**). Higher expression levels of ADAR1, CALR and β-catenin proteins were found in peritoneal metastasis than gastric cancer tissues (**Figure 2G-I**).

### Co-localization of ADAR1 with CALR, Wnt / β-catenin pathway- and EMT-related proteins in gastric cancer peritoneal metastasis

**Figure 3:**
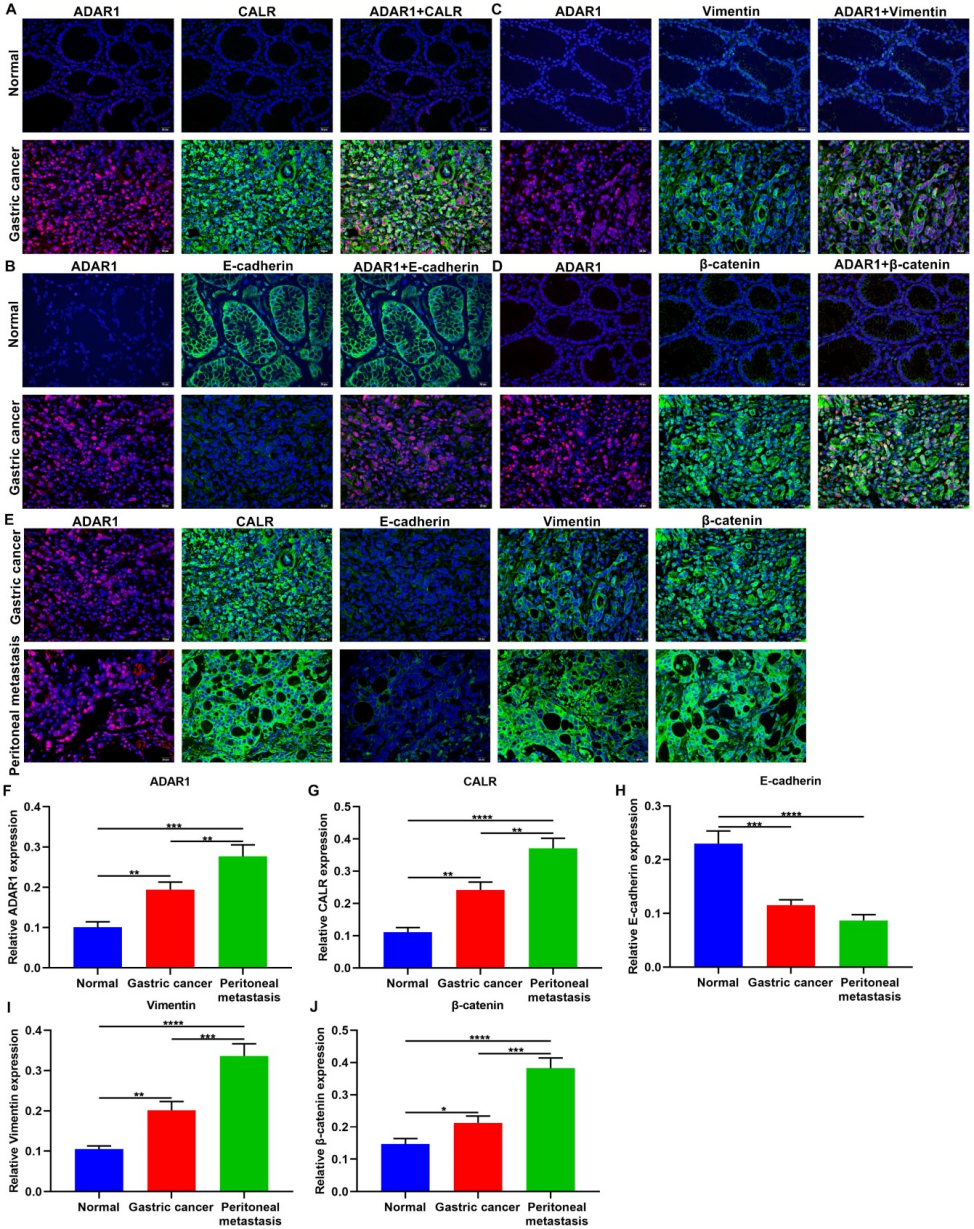
Co-localization of ADAR1 with CALR and Wnt / β-catenin pathway and EMT proteins in gastric cancer peritoneal metastasis. Immunofluorescence for the co-localization of ADAR1 with (A) CALR, (B) E-cadherin, (C) Vimentin, and (D) β-catenin proteins in normal and gastric cancer tissues. (E) Immunofluorescence for the expression of ADAR1, CALR, E-cadherin, Vimentin, and β-catenin proteins in gastric cancer and peritoneal metastasis tissues. Quantification of the expression of (F) ADAR1, (G) CALR, (H) E-cadherin, (I) Vimentin, and (J) β-catenin proteins in normal, gastric cancer and peritoneal metastasis tissues. Bar = 20 μm. *p<0.05; **p<0.01; ***p<0.001; ****p<0.0001.

Immunofluorescence results confirmed the co-localization of ADAR1 with CALR, E-cadherin, Vimentin, and β-catenin proteins in normal and gastric cancer tissue samples (**Figure 3A-D**). We found that ADAR1 was mainly expressed in the nucleus in gastric cancer tissues, while CALR was primarily distributed in the cell cytoplasm and membrane in gastric cancer tissues (**Figure 3A**). In **Figure 3B**, E-cadherin was mainly expressed in the plasma membrane, extracellular and cytoskeleton in normal tissues. Meanwhile, Vimentin was principally expressed in the cell cytoplasm of gastric cancer tissues (**Figure 3C**). For β-catenin, it was mainly distributed in the nucleus and cytoplasm in gastric cancer tissues (**Figure 3D**). Also, we investigated their localization in gastric cancer and peritoneal metastasis tissues (**Figure 3E**). After quantification, ADAR1 and CALR were both highly expressed in gastric cancer than normal tissues (**Figure 3F, G**). Higher ADAR1 and CALR expression was distinctly detected in peritoneal metastasis than primary tumor tissues. E-cadherin displayed lowered expression in gastric cancer than normal tissues (**Figure 3H**). Meanwhile, its expression was markedly decreased in peritoneal metastasis than primary tumor tissues. There were significantly higher Vimentin and β-catenin levels in gastric cancer compared to normal tissues (**Figure 3I, J**). Furthermore, Vimentin and β-catenin exhibited elevated expression levels in peritoneal metastasis than primary tumor tissues. These data indicated that ADAR1, CALR, Wnt / β-catenin pathway and EMT process proteins might be related to gastric cancer peritoneal metastasis.

### Targeting ADAR1 suppresses peritoneal metastasis of gastric cancer in mouse models

**Figure 4:**
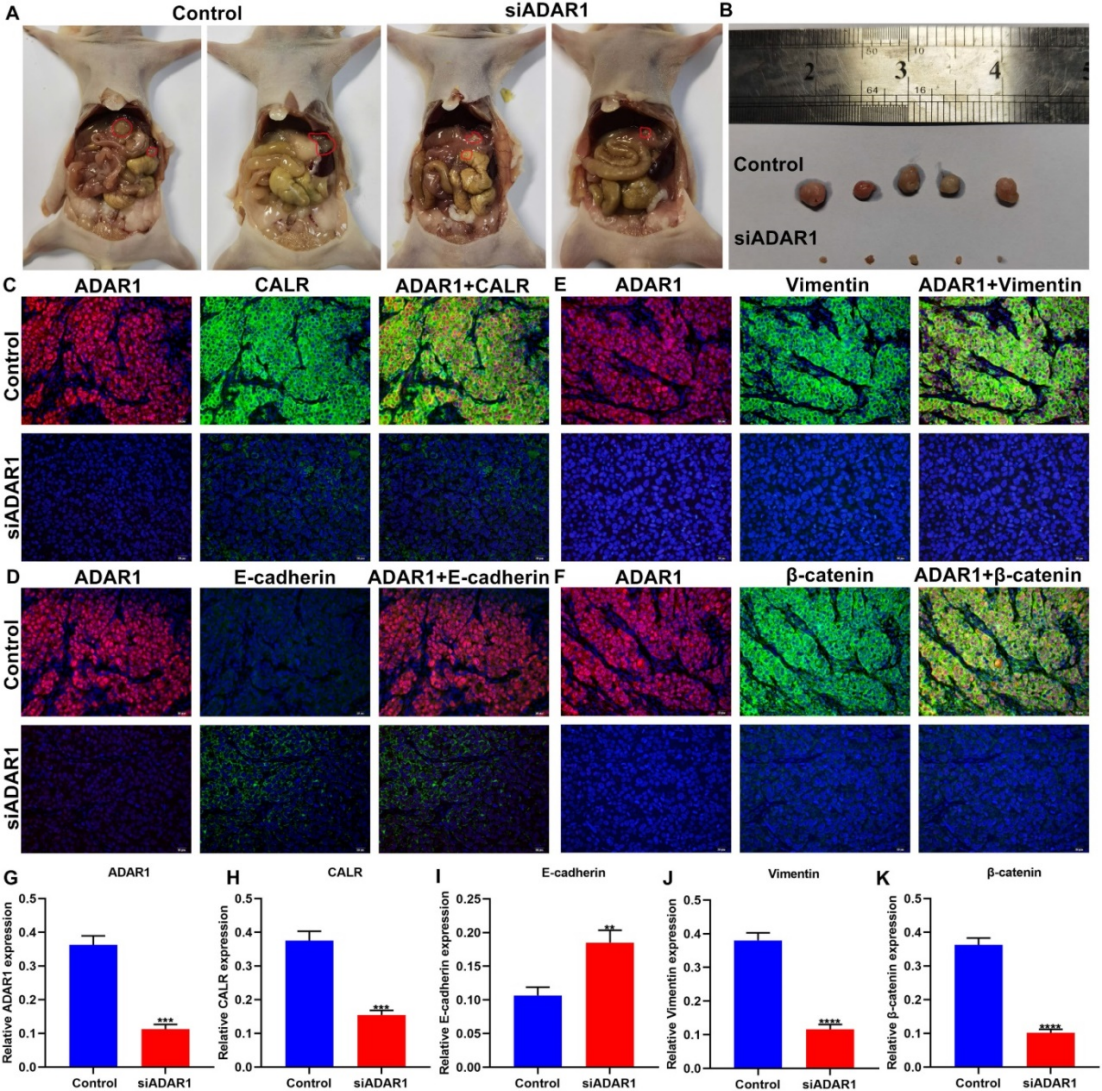
Si-ADAR1 treatment ameliorates peritoneal metastasis of gastric cancer *in vivo*. (A) Representative graphs of gastric cancer peritoneal metastasis models in nude mice. There were 5 nude mice in each group. (B) Peritoneal metastatic tumors of control and si-ADAR1 groups. Immunofluorescence for the co-localization of ADAR1 with (C) CALR, (D) E-cadherin, (E) Vimentin, and (F) β-catenin proteins in peritoneal metastatic tumor tissues. Bar = 20 μm. Quantitative results of (G) ADAR1, (H) CALR, (I) E-cadherin, (J) Vimentin, and (K) β-catenin expression in peritoneal metastatic tumors of control and si-ADAR1 groups. **p<0.01; ***p<0.001; ****p<0.0001.

Here, we established a peritoneal metastasis model of gastric cancer in nude mice. Following treatment with si-ADAR1 for 4 weeks, we removed the tumors in the abdominal cavity of models. Our data showed that si-ADAR1 treatment distinctly decreased the volume of intraperitoneal metastatic tumors (**Figure 4A, B**). Immunofluorescence was presented for evaluating the expression of ADAR1, CALR, E-cadherin, Vimentin, and β-catenin proteins in peritoneal metastatic tumor tissues (**Figure 4C-F**). Compared to control group, ADAR1 expression was significantly reduced in peritoneal metastatic tumor tissues of si-ADAR1 group (**Figure 4G**). Furthermore, lowered CALR expression was detected in peritoneal metastatic tumors following treatment with si-ADAR1 (**Figure 4H**). In **Figure 4I**, targeting ADAR1 elevated E-cadherin expression in peritoneal metastatic gastric cancer tissues. In comparison to control group, the expression levels of Vimentin and β-catenin were significantly reduced in peritoneal metastatic tumors of si-ADAR1 group (**Figure 4J, K**). Collectively, targeting ADAR1 treatment suppressed peritoneal metastasis of gastric cancer.

### Targeting ADAR1 decreases gastric cancer invasion and metastasis in mouse models

**Figure 5:**
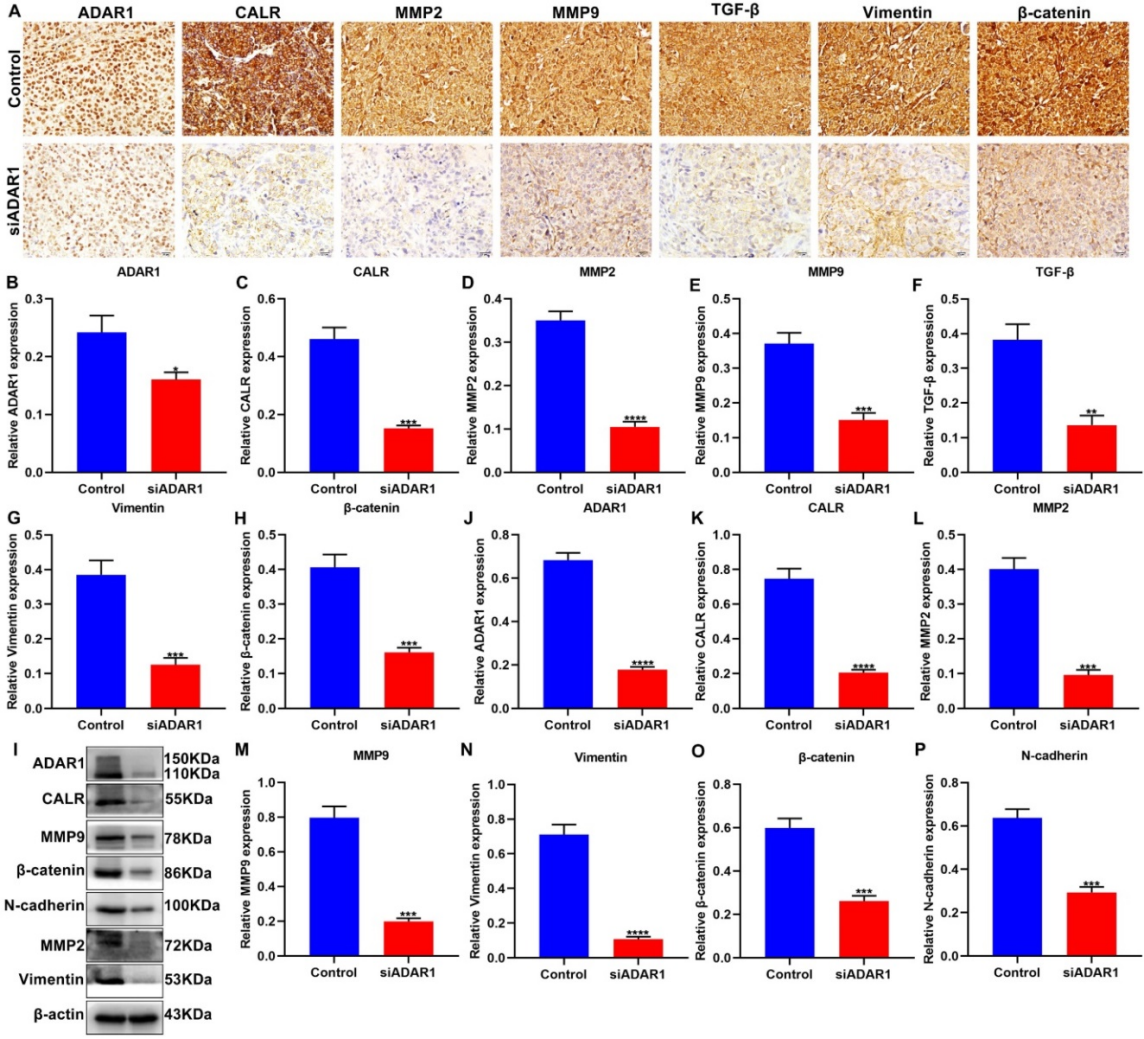
Targeting ADAR1 decreases invasion and metastasis of gastric cancer *in vivo*. (A) Immunohistochemistry for the expression of (B) ADAR1, (C) CALR, (D) MMP2, (E) MMP9, (F) TGF-β, (G) Vimentin and (H) β-catenin in peritoneal metastatic tissues of nude mice from control and si-ADAR1 groups. Bar = 20 μm. (I) Western blot for detecting the expression of (J) ADAR1, (K) CALR, (L) MMP2, (M) MMP9, (N) Vimentin, (O) β-catenin and (P) N-cadherin in peritoneal metastatic tissues of mouse models. *p<0.05; **p<0.01; ***p<0.001; ****p<0.0001.

We further performed immunohistochemistry to detect ADAR1 and CALR expression in gastric cancer peritoneal metastatic tissues of nude mice. As expected, the expression of ADAR1 and CALR exhibited significant reduction in si-ADAR1 group than control group (**Figure 5A-C**). MMPs facilitate invasion and metastasis of tumor cells via digesting extracellular matrix (ECM) 22. It has been found that the family members of MMPs like MMP2 and MMP9 are up-regulated in human metastatic gastric cancer 22. We found that, compared to control group, MMP2 and MMP9 expression was both decreased in peritoneal metastatic tissues of si-ADAR1 group (**Figure 5D, E**). Furthermore, targeting ADAR1 significantly decreased the expression of TGF-β, Vimentin and β-catenin proteins than controls (**Figure 5F-H**). The expression of above proteins in peritoneal metastatic tissues was also detected via western blot (**Figure 5I**). with one accord, the expression levels of ADAR1 (**Figure 5J**), CALR (**Figure 5K**), MMP2 (**Figure 5L**), MMP9 (**Figure 5M**), Vimentin (**Figure 5N**), β-catenin (**Figure 5O**) and N-cadherin (**Figure 5P**) proteins in peritoneal metastatic tissues were significantly reduced after treatment with si-ADAR1. These findings indicated that targeting ADAR1 may decrease invasion and metastasis of gastric cancer.

### ADAR1 knockdown restrains proliferation of gastric cancer cells partly by Wnt / β-catenin pathway

**Figure 6:**
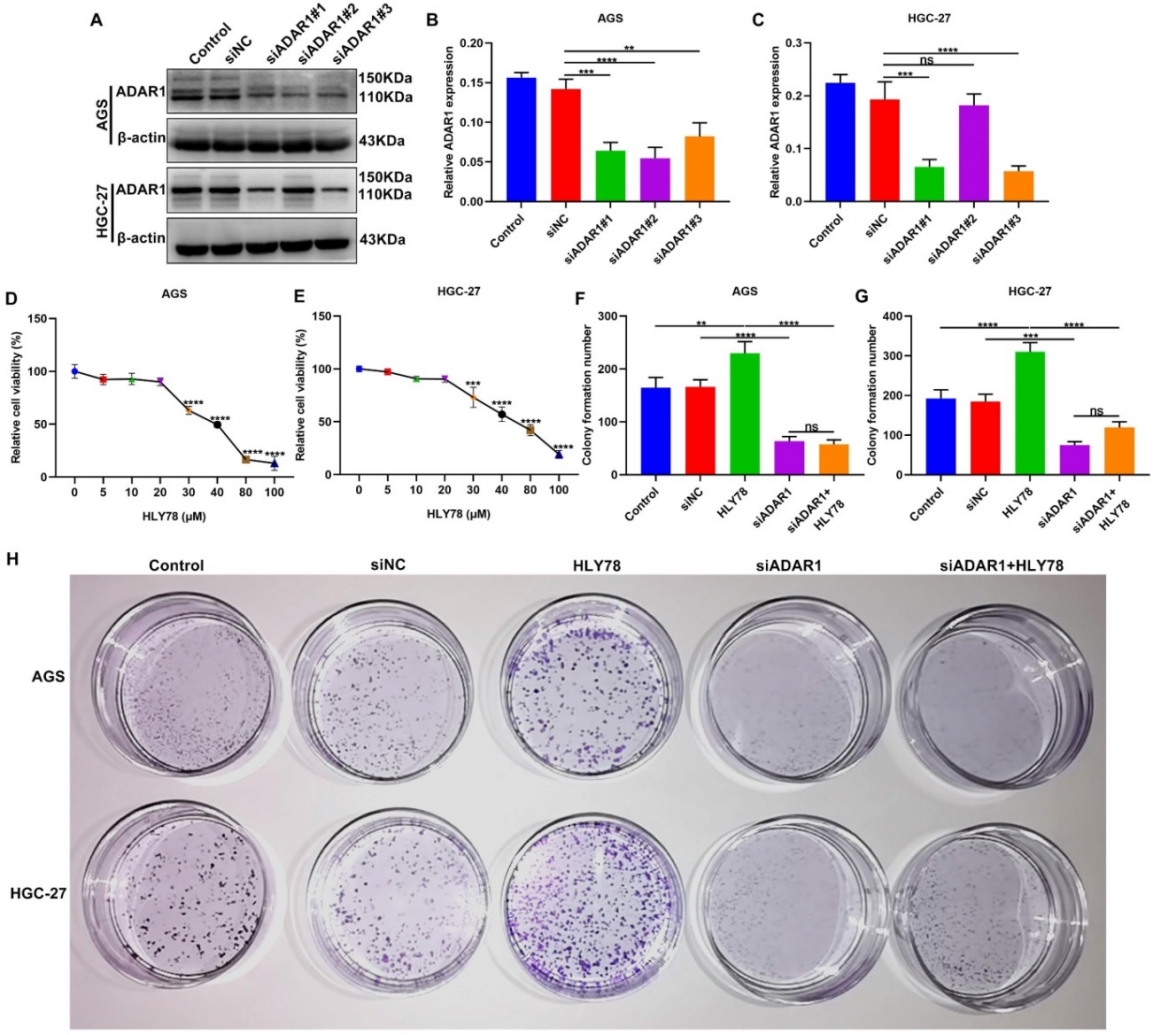
ADAR1 knockdown suppresses proliferation of gastric cancer cells partly by Wnt / β-catenin pathway. (A-C) Western blot for ADAR1 expression in AGS and HGC-27 cells transfected with three siRNAs against ADAR1. (D, E) CCK-8 for cell viability of AGS and HGC-27 cells treated with a series of concentrations of HLY78. (F-H) Colony formation assay of AGS and HGC-27 cells under treatment with si-ADAR1 and / or HLY78. **p<0.01; ***p<0.001; ****p<0.0001; ns: not significant.

To observe the functions of ADAR1 on gastric cancer progression, three siRNAs against ADAR1 were transfected into AGS and HGC-27 cells. Western blot confirmed that ADAR1 expression was significantly reduced by si-ADAR1 (**Figure 6A-C**). Here, si-ADAR1#1 exhibited the best transfection effects. Thus, we chose si-ADAR1#1 for further experiments. *In vivo*, targeting ADAR1 suppressed the expression of proteins in the Wnt / β-catenin pathway. In this study Wnt activator HLY78 was utilized for activation of this pathway. To determine the optimal concentration of HLY78, CCK-8 assay was performed to examine cell viability of AGS and HGC-27 cells treated with a series of concentrations of HLY78. Our data showed that when the concentration of HLY78 reached 30 μM, distinct cytotoxicity appeared in AGS and HGC-27 cells (**Figure 6D, E**). Hence, 20 μM HLY78 was chosen as the optimal concentration. As shown in colony formation assay, si-ADAR1 transfection significantly reduced the number of colony formation in AGS and HGC-27 cells (**Figure 6F-H**). On the contrary, colony formation ability was distinctly enhanced by HLY78 treatment. However, ADAR1 knockdown significantly weakened the enhancement of colony formation ability induced by HLY78 treatment. But HLY78 did not change the inhibitory effects of si-ADAR1 on colony formation of gastric cancer cells. These data indicated that targeting ADAR1 restrained proliferation of gastric cancer cells partly by suppressing Wnt / β-catenin pathway.

### Silencing ADAR1 lessens migration of gastric cancer cells partly via Wnt / β-catenin pathway

**Figure 7:**
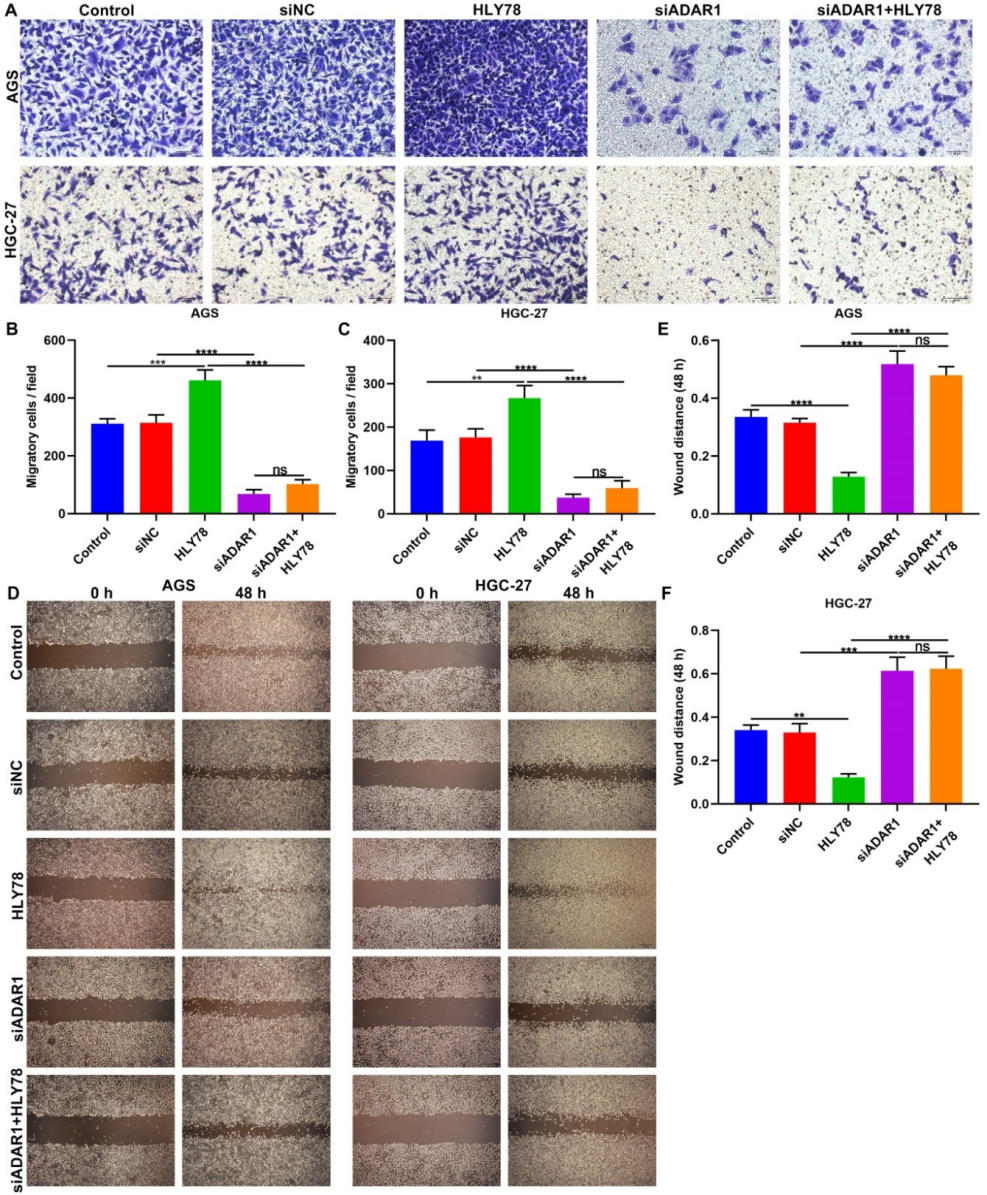
Silencing ADAR1 restrains migration of gastric cancer cells partly by Wnt / β-catenin pathway. (A-C) Transwell for the number of migratory AGS and HGC-27 cells under treatment with HLY78 and / or si-ADAR1. Bar = 50 μm. (D-F) Wound healing for the wound distance of AGS and HGC-27 cells treated with HLY78 and / or si-ADAR1. Bar = 200 μm. **p<0.01; ***p<0.001; ****p<0.0001; ns: not significant.

The functions of ADAR1 on migration of gastric cancer cells were assessed in depth. We found that si-ADAR1 significantly reduced the number of migratory cells in AGS and HGC-27 cells (**Figure 7A-C**). Wnt activator HLY78 significantly facilitated migration of gastric cancer cells, which was distinctly weakened by si-ADAR1 co-treatment. Nevertheless, HLY78 did not affect the inhibitory functions of si-ADAR1 on migration. Migratory ability of gastric cancer cells was also investigated by wound healing assay. As a result, the wound distance of AGS and HGC-27 cells was significantly widened by ADAR1 knockdown (**Figure 7D-F**). Oppositely, HLY78 treatment distinctly shortened the wound distance of gastric cancer cells. But silencing ADAR1 markedly receded the enhancement on migration induced by HLY78. HLY78 co-treatment did not change the suppressive roles of ADAR1 knockdown on migration of gastric cancer cells. Taken together, silencing ADAR1 lessened migratory capacities of gastric cancer cells partly through suppressing Wnt / β-catenin pathway.

### ADAR1 knockdown inhibits CALR expression, Wnt / β-catenin pathway and EMT process in gastric cancer cells

**Figure 8:**
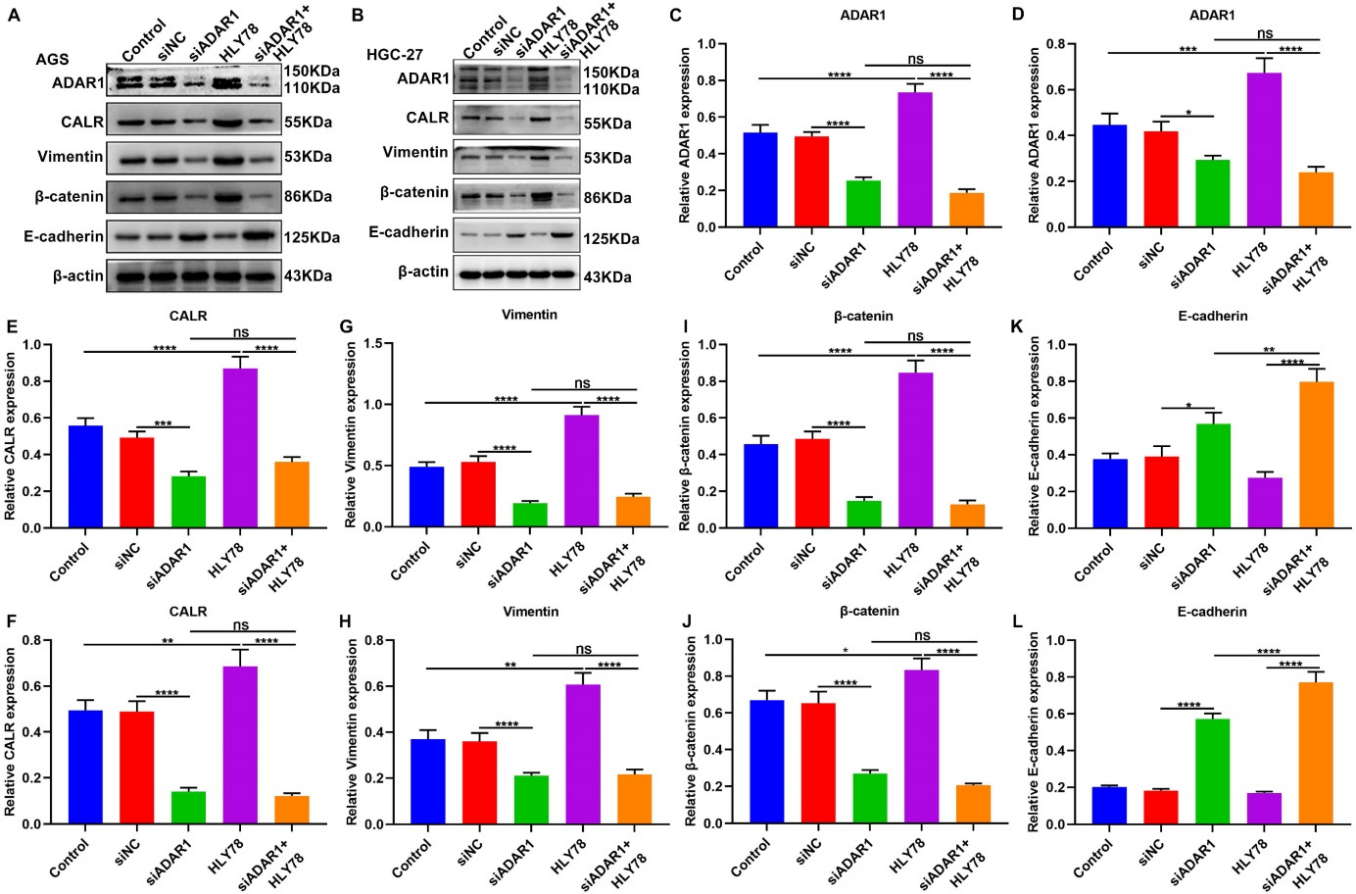
Silencing ADAR1 inhibits CALR expression, Wnt / β-catenin pathway and EMT process in gastric cancer cells. (A, B) Western blot for the expression of (C, D) ADAR1, (E, F) CALR, (G, H) Vimentin, (I, J) β-catenin and (K, L) E-cadherin in AGS and HGC-27 cells treated with HLY78 and / or si-ADAR1. *p<0.05; **p<0.01; ***p<0.001; ****p<0.0001; ns: not significant.

We performed western blot to observe the effects of ADAR1 knockdown on CALR expression, Wnt / β-catenin pathway and EMT process in gastric cancer cells (**Figure 8A, B**). Our data showed that HLY78 treatment distinctly increased ADAR1 expression in AGS and HGC-27 cells, which was weakened by ADAR1 knockdown (**Figure 8C, D**). But HLY78 did not affect ADAR1 expression under transfection with si-ADAR1. Furthermore, we found that ADAR1 knockdown distinctly decreased the expression of CALR (**Figure 8E, F**), Vimentin (**Figure 8G, H**) and β-catenin (**Figure 8I, J**) in AGS and HGC-27 cells, while their expression was significantly increased by HLY78 treatment. ADAR1 knockdown distinctly ameliorated the increase in CALR, Vimentin and β-catenin expression induced by HLY78. However, the inhibitory effects of ADAR1 knockdown on their expression were not affected by HLY78 treatment. In **Figure 8K, L**, E-cadherin expression was elevated by ADAR1 knockdown in AGS and HGC-27 cells. HLY78 suppressed its expression, which was not statistically significant. Collectively, ADAR1 knockdown suppressed CALR expression, Wnt / β-catenin pathway as well as EMT process in gastric cancer cells.

### 8-Azaadenosine ADAR1 inhibitor suppresses proliferation of gastric cancer cells

**Figure 9:**
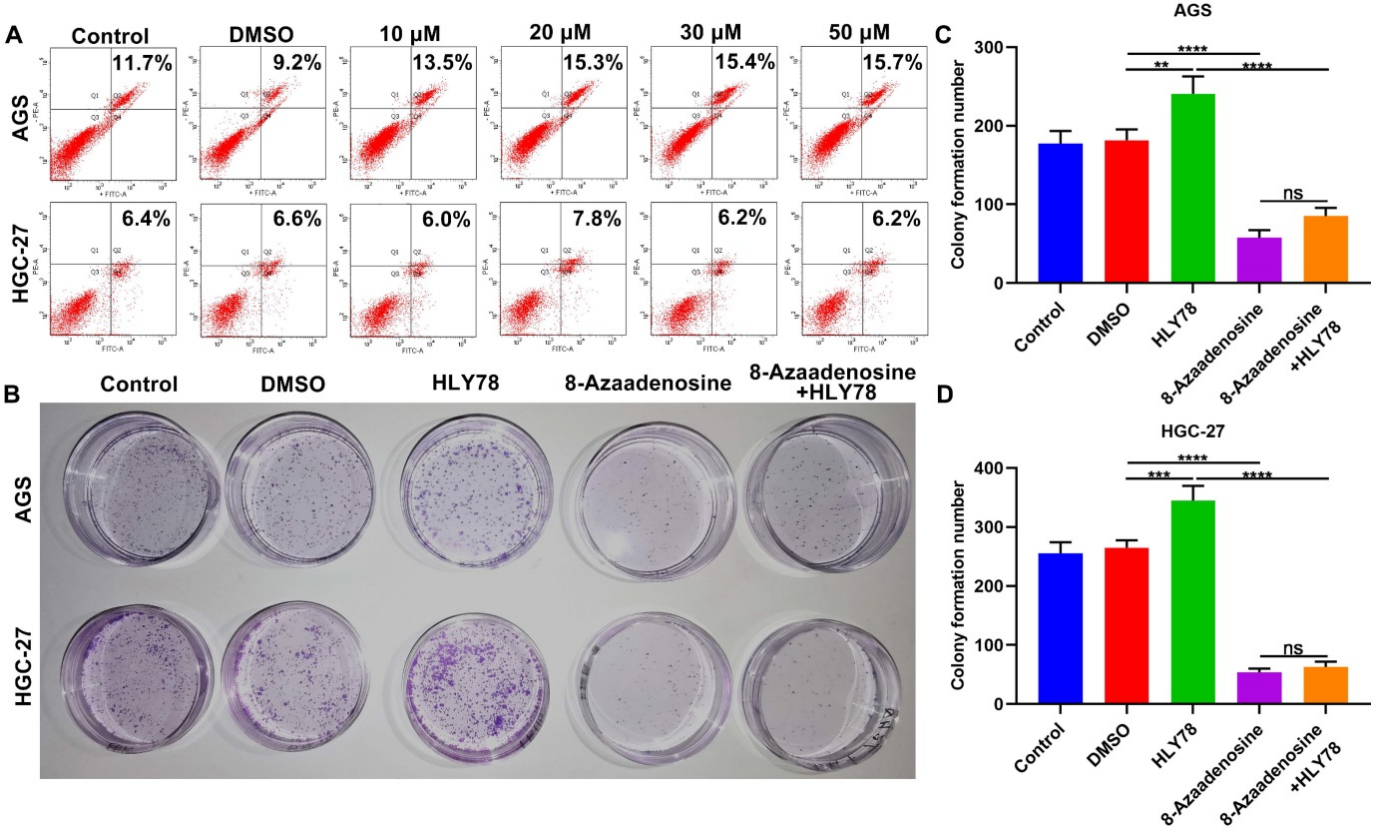
8-Azaadenosine ADAR1 inhibitor restrains proliferation of gastric cancer cells. (A) Flow cytometry for apoptosis of AGS and HGC-27 cells treated with a series of concentrations of 8-Azaadenosine. (B-D) Colony formation assay for the proliferation of AGS and HGC-27 cells treated with 8-Azaadenosine and / or HLY78. **p<0.01; ***p<0.001; ****p<0.0001; ns: not significant.

The ADAR1 mainly exerts a role on A-to-I editing of RNA that is the most frequent editing form in humans 23. Here, we observed whether ADAR1 was involved in oncogenic processes by RNA editing. 8-Azaadenosine, as an ADAR1 inhibitor, acts on double-stranded RNAs. Flow cytometry was utilized to identify the optimal dosage of 8-Azaadenosine on gastric cancer cells. AGS and HGC-27 cells were treated with a series of concentrations of 8-Azaadenosine (**Figure 9A**). Herein, 10 μM 8-Azaadenosine was chosen for colony formation assay. As a result, 8-Azaadenosine treatment distinctly lessened the number of colony formation of AGS and HGC-27 cells (**Figure 9B-D**). Furthermore, 8-Azaadenosine markedly weakened the increase in colony formation number induced by HLY78. But HLY78 did not change the suppressive roles of 8-Azaadenosine on proliferation of AGS and HGC-27 cells. These data were indicative that ADAR1-mediated A-to-I editing of RNA could facilitate gastric cancer progression.

### Targeting ADAR1 suppresses CALR and β-catenin expression in gastric cancer

**Figure 10:**
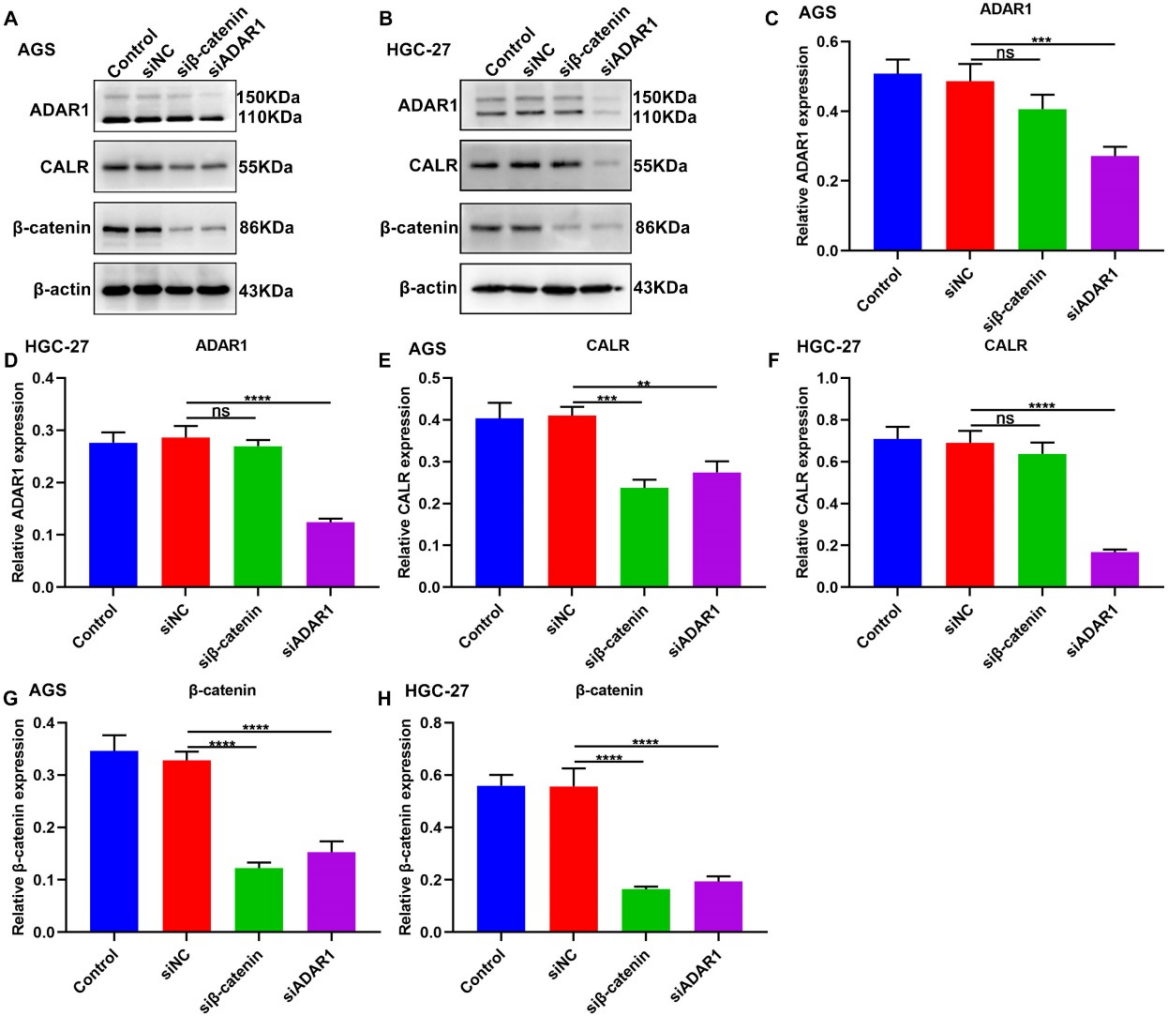
Targeting ADAR1 inhibits CALR and β-catenin expression in gastric cancer. (A, B) Western blot for the expression of (C, D) ADAR1, (E, F) CALR and (G, H) β-catenin proteins in AGS and HGC-27 cells transfected with siRNAs against β-catenin or ADAR1. **p<0.01; ***p<0.001; ****p<0.0001; ns: not significant.

We further observed the roles of ADAR1 on CALR and β-catenin in gastric cancer by western blot (**Figure 10A, B**). Firstly, β-catenin knockdown did not change ADAR1 expression in AGS and HGC-27 cells (**Figure 10C, D**). But silencing β-catenin significantly decreased CALR expression in AGS (**Figure 10E**) not HGC-27 cells (**Figure 10F**). The inhibitory roles of ADAR1 knockdown on CALR expression were observed in AGS and HGC-27 cells (**Figure 10E, F**). Furthermore, we found that silencing ADAR1 or β-catenin markedly lessened β-catenin expression both in AGS and HGC-27 cells (**Figure 10G, H**). Thus, targeting ADAR1 suppressed CALR and β-catenin expression in gastric cancer.

## Discussion

This study identified a novel tumorigenic role of ADAR1 on peritoneal metastasis of gastric cancer. Our data confirmed that targeting ADAR1 distinctly suppressed gastric cancer metastasis. Mechanically, ADAR1 knockdown distinctly reduced oncogene CALR expression as well as inactivated Wnt / β-catenin pathway in gastric cancer cells. These findings demonstrated that ADAR1 could be a promising therapeutic target against gastric cancer metastasis.

Patients with peritoneal metastasis of gastric cancer exhibit dismal survival outcomes 24. In our cohort, ADAR1 up-regulation displayed a significant correlation to depth of invasion, TNM stage as well as peritoneal metastasis, as previously reported 14. To verify the functions of ADAR1 on peritoneal metastasis of gastric cancer, we established a Xenograft nude mouse model. siADAR1 treatment markedly reduced the volume of peritoneal metastatic tumors, highlighting the tumorigenic role of ADAR1 on peritoneal metastasis of gastric cancer. Consistently with previous research, CALR was overexpressed in gastric cancer, and its up-regulation was distinctly correlated to lymph node metastasis and peritoneal metastasis 25. CALR up-regulation significantly reinforced angiogenesis and metastasis in gastric cancer 26. ADAR1 knockdown markedly suppressed CALR expression, indicating that CALR was a downstream target of ADAR1. EMT is a key pathway for tumor cell invasion and metastasis 27. Down-regulation of E-cadherin expression and up-regulation of vimentin expression are the main characteristics of the EMT process 28. Our data demonstrated that silencing ADAR1 inactivated EMT process in gastric cancer cells. Furthermore, ADAR1 knockdown weakened proliferation and migration of gastric cancer cells. As previously reported, ADAR1 could mediate melanoma invasion and metastasis via controlling ITGB3 expression 29. ADAR1 p110 facilitates adhesion of liver cancer cells to extracellular matrix through elevating ITGA2 30. The above results illustrate the carcinogenesis of ADAR1 on gastric cancer metastasis.

Our research found that ADAR1 could promote Wnt/β-catenin pathway in gastric cancer cells. Silencing ADAR1 can inhibit β-catenin protein levels. As a previous study, ADAR1 may elevate Wnt pathway in acute myeloid leukemia cells 31. Administration of Wnt/β-catenin pathway agonist can promote the proliferation and migration of gastric cancer cells, reduce the levels of N-cadherin, Vimentin, and β-catenin proteins, and increase the levels of E-cadherin proteins. But ADAR1 knockdown could reverse the activation in EMT process, proliferation and migration induced by Wnt/β-catenin agonist, while this agonist did not change the inhibitory effects of ADAR1 knockdown on migration and metastasis of gastric cancer cells. Also, silencing ADAR1 markedly reduced β-catenin expression but β-catenin knockdown did not affect ADAR1 expression in gastric cancer cells. Hence, ADAR1 may facilitate gastric cancer metastasis by Wnt / β-catenin pathway.

It has been found that ADAR1-mediated RNA editing facilitates gastric cancer progression 32. Herein, gastric cancer cells were treated with 8-Azaadenosine ADAR1 inhibitor that acted on double-stranded RNAs. Our data suggested that 8-Azaadenosine restrained proliferation of gastric cancer cells, indicating that targeting ADAR-mediated RNA editing could suppress gastric cancer progression. Several limitations of this study should be pointed out. Firstly, although our study confirmed that silencing ADAR1 could inhibit gastric cancer metastasis in nude mouse models, more preclinical research requires to confirm the effects of ADAR1 on gastric cancer metastasis. Secondly, the value of ADAR1 in predicting gastric cancer metastasis should be validated in a larger prospective cohort. Thirdly, the mechanisms of ADAR1 on gastric cancer metastasis will be further investigated in our future studies.

## Conclusion

Collectively, this study provided novel evidence of the roles of ADAR1 on peritoneal metastasis of gastric cancer. Silencing ADAR1 weakened metastatic potential of gastric cancer cells. Thus, ADAR1 may be a therapeutic target for gastric cancer metastasis.

## Figures and Tables

**Table 1:** Correlation between ADAR1 expression and clinicopathologic features across gastric cancer patients.

Clinical parameters	Total (n=95)	ADAR1		Chi-square	P-value
		Positive (68)	Negative (27)		
Gender					
Male	57	44	13	2.208	0.137
Female	38	24	14		
Age					
<60	63	42	21	2.218	0.136
≥60	32	26	6		
Depth of invasion					
T1/T2	38	21	17	8.287	0.004**
T3/T4	57	47	10		
Lymph metastasis					
N0-1	17	10	7	1.656	0.198
N2-3	78	58	20		
TNM stage					
Ⅰ-Ⅱ	52	27	25	21.818	0.000***
Ⅲ-Ⅳ	43	41	2		
Peritoneal metastasis					
Absent	63	40	23	6.012	0.014*
Present	32	28	4		

*P<0.05; **P<0.01; ***P<0.001.

## Abbreviations

EMT: epithelial-mesenchymal transition
ADAR1: adenosine deaminase 1
IFN: interferon
CCK-8: Cell counting kit-8
TCGA: The Cancer Genome Atlas
